# Clinical implications of posterior semicircular canal function in idiopathic sudden sensorineural hearing loss

**DOI:** 10.1038/s41598-020-65294-5

**Published:** 2020-05-20

**Authors:** Hayoung Byun, Jae Ho Chung, Seung Hwan Lee

**Affiliations:** Department of Otolaryngology-Head and Neck Surgery. College of Medicine, Hanyang University, Seoul, Republic of Korea

**Keywords:** Risk factors, Signs and symptoms, Prognostic markers, Neurological disorders

## Abstract

Predicting hearing outcomes in idiopathic sudden sensorineural hearing loss (ISSNHL) is still challenging. We hypothesized that assessment of the semicircular canal (SCC) function via the video head impulse test (vHIT) might provide prognostic information. The medical records of patients diagnosed with ISSNHL from January 2015 to December 2018 were retrospectively reviewed. The prognostic values of the vHIT and other previously known factors in predicting hearing recovery were analyzed using a logistic regression model. A total of 148 patients with normal contra-lesional hearing were analyzed. Fifty-seven patients exhibited low gain (<0.7) on the vHIT in at least one SCC, more than the number of patients complaining of dizziness. Multivariable analysis revealed that non-recovery of normal hearing was associated with older age (OR 1.040), worse canal paresis on the caloric test (OR 1.023), worse initial hearing thresholds (OR 1.045) and abnormal vHIT result in the posterior SCC (OR 3.670). Low vHIT gain in the posterior SCC had specificity of 94.4% and positive predictive value of 85.7% in predicting non-recovery of normal hearing. In conclusion, abnormal vHIT gain in the posterior SCC appears to be a specific prognostic factor for incomplete hearing recovery in ISSNHL.

## Introduction

Sudden sensorineural hearing loss (SSNHL) is defined as sensorineural hearing loss of 30 dB or more over at least three consecutive frequencies occurring within 72 hours^1,2^. In the majority of patients there is no specific identifiable cause of the hearing loss, and these cases are classified as idiopathic SSNHL (ISSNHL)^1^. Various etiologic backgrounds have been proposed, such as viral infection, vascular insufficiency and immunologic reaction^3,4^. ISSNHL has been treated with empirical therapies such as high dose steroid, antivirals, hyperbaric oxygen, etc^1,5^. Even if managed promptly, the outcomes of ISSNHL vary from non-recovery of previous hearing to complete recovery.

**Figure 1:**
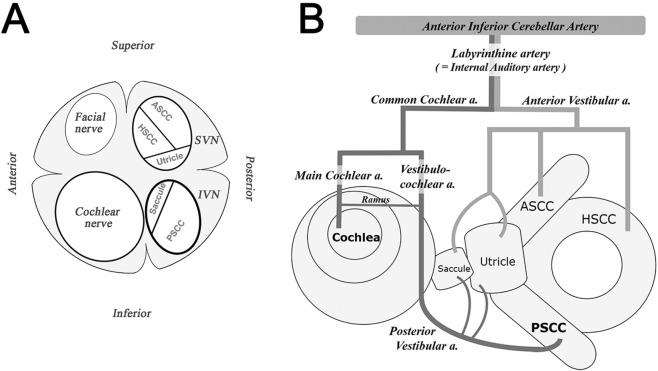
Schematic drawing of a transverse section through the internal auditory canal (**A**) showing the relationships between the facial nerve, the cochlear nerve and the vestibular nerves innervating the saccule, utricle and semicircular canals. Simplified drawing of the blood supply to the inner ear (**B**). SVN; superior vestibular nerve, IVN; inferior vestibular nerve, ASCC; anterior semicircular canal, HSCC; horizontal semicircular canal, PSCC; posterior semicircular canal, a.; artery.

One of the most challenging aspects of ISSNHL for both clinician and patient is its uncertainty in terms of etiology, prognosis and treatment. It is difficult to predict hearing outcomes, as well as to identify the causes of individual hearing loss. Efforts have been made to establish prognoses, and known prognostic indicators include severity of initial hearing loss, age at onset, presence of vertigo, shape of audiogram and early treatment^1^. The prevalence of dizziness or imbalance in SSNHL has been reported to be about 30%, and is related to poor prognosis^1,4^. Because vertigo is a subjective symptom, unlike the other indicators, a variety of studies have been performed to elucidate the clinical significance of vestibular function tests in SSNHL. Previous reports have focused on the value of the caloric test and vestibular evoked myogenic potentials (VEMP), which reflect the functioning of the horizontal semicircular canals (SCC) and otolith organs^6–9^. Theoretically, however, various types of vestibulocochlear involvement can be expected in ISSNHL. In terms of anatomic position, the closest vestibular organ from the cochlea is the saccule. The endolymphatic fluid spaces of cochlea and saccule are connected via ductus reunions, and their innervating nerve fibers are close together (Fig. 1A). In terms of blood supply of the inner ear, the anterior vestibular branch of the labyrinthine artery feeds all the vestibular organs except the posterior SCC and part of the saccular macula, which are supplied by branches of the common cochlear artery – the posterior vestibular artery - without collaterals (Fig. 1B)^10,11^.

Accordingly, we aimed to evaluate localized dysfunction of the vestibular organ in ISSNHL. We hypothesized that each of the components of the vestibular organ, especially the SCCs, can be involved variously in ISSNHL and might have distinct prognostic implications. Since subjective symptoms are not always correlated with objective vestibular function test results^9^, we evaluated all ISSNHL patients, including those who did not complain of dizziness. The functioning of each SCC and saccule was assessed using the video Head Impulse Test (vHIT), bi-thermal caloric test and air-conducted cervical VEMP. The aim of the study was to evaluate the clinical significance of SCC involvement, as assessed by the vHIT, in predicting hearing outcomes of ISSNHL.

## Materials and Methods

### Patients and study design

The medical records of patients diagnosed with unilateral ISSNHL from January 2015 to December 2018 were retrospectively reviewed. The diagnosis of SSNHL was based on sudden hearing loss of more than 30 dB at a minimum of 3 consecutive frequencies over a period of 72 hours or less^12^. At the time of diagnosis, otoendoscopic examination, audiometry and vestibular function tests including the caloric test, vHIT and cVEMP, were routinely performed in all patients. Subjects with pre-existing hearing loss in the contra-lesional normal ear exceeding 25 dB HL were excluded to avoid possible debate about the assessment of outcomes. Those with retrocochlear pathology or other ear diseases - vestibular schwannoma, Meniere’s disease, inner ear anomaly, perilymphatic fistula, conductive hearing loss, or AICA infarction on MRI - were also excluded.

According to the uniform treatment protocol, patients were initially treated with high dose oral steroid (prednisone 1 mg/kg daily for 7 days followed by 4 days of tapering). Thereafter, salvage intratympanic dexamethasone (5 mg/mL) injections (4 times/2 weeks) were performed in those whose hearing thresholds did not reach serviceable level (<40 dB HL) within a week.

Recovery of hearing was determined at 3 months from onset according to Siegel’s criterion, which is widely used to report hearing gain in SSNHL^13^. Complete recovery was defined as a final hearing threshold <25 dB HL. Partial recovery was defined as a final hearing threshold of 26–45 dB HL and >15 dB of hearing gain. Slight improvement referred to a final hearing threshold >46 dB HL and <than 15 dB of hearing gain, and no improvement meant a final hearing threshold >76 dB HL or <15 dB of hearing gain.

In this study, the complete recovery group according to Siegel’s criterion, namely patients with final hearing threshold <25 dB HL, was the group of interest. The other groups were combined as the incomplete recovery group.

### Ethical issues

This investigation was approved by the ethics review board of Hanyang University Guri Hospital (IRB #2019-11-016) and performed in accordance with the Declaration of Helsinki and good clinical practice guidelines. Informed consent was waived because of the retrospective nature of the study, and the analysis used anonymous clinical data after approval of ethics review board of Hanyang University Guri Hospital.

### The video head impulse test

The video head impulse test (vHIT) was performed at presentation to evaluate the high-frequency vestibulo-ocular reflex (VOR) in each SCC plane, using an ICS Impulse (GN Otometrics, Taastrup, Denmark). To enhance test reliability, two experienced examiners performed the test, using the standard protocol proposed by Halmagy^14^. Head impulses were given at least 10 times at the low amplitude of 10 degrees and a consistent peak velocity (100–300/second). Eye and head velocities were recorded. A calculated VOR gain of <0.7 was considered abnormal.

### The caloric and cVEMP tests

The bi-thermal caloric test was performed using binaural alternative instillation of 8 liters of cold (24 °C) and warm (50 °C) air for 60 seconds^15,16^. The induced nystagmus was recorded by video-nystagmography (ICS Medical, Schaumburg, IL, USA) until it had decayed to the null position. The maximum slow phase velocities of the corresponding stimuli were assessed and the asymmetry of vestibular function was calculated using Jongkees’ formula^15,17^. Canal paresis >25% was defined as horizontal semi-circular dysfunction^16,18^.

The cVEMP test was performed with a Biologic Navigator Pro (Biologic System Corp., IL, USA). The reference electrode, ground electrode and two active electrodes were placed at the sternoclavicular notch, center of the forehead and the middle third of each sternocleidomastoid muscle, respectively^19,20^. Using a tone-burst sound stimulus of 500 Hz and 90 dB nHL via an EA-3 insert ear phone, the amplitudes of evoked potentials (P13-N23) on the same side of the sternocleidomastoid muscle were recorded^19,20^. cVEMP abnormality was defined as an asymmetry ratio of p13-n23 amplitude >35%, or no significant P13-N23 waveform^19–21^.

### Statistical analysis

Data were analyzed with IBM SPSS Statistics, version 24.0 for Windows (IBM Corp., NY, USA). Descriptive data are expressed as means and standard deviations. A logistic regression model was used to evaluate the effects of prognostic factors on incomplete hearing recovery. Each parameter was assessed by univariable logistic regression analysis, and statistically significant factors were included in a subsequent multivariable analysis. The sensitivity, specificity, positive predictive value (PPV) and negative predictive value (NPV) of the vHIT for predicting non-recovery of normal hearing – incomplete recovery – were calculated. P values <0.05 were considered to indicate statistical significance. Figures were drawn with Microsoft Powerpoint 2016 MSO (Microsoft Corp., WA, USA).

## Results

### Demographics

**Figure 2:**
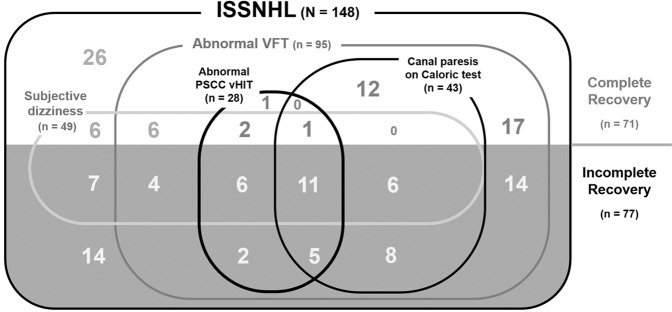
Venn diagram of the relationships between subjective dizziness, vestibular function test results and hearing outcomes. ISSNHL; idiopathic sudden sensorineural hearing loss, VFT; vestibular function test, PSCC; posterior semicircular canal, vHIT; video head impulse test.

A total of 148 consecutive patients (mean age 49.7 ± 13.9 years) with ISSNHL were analyzed. The average initial hearing threshold was 62.2 ± 29.5 dB HL (Table 1). Of the 148 patients, 49 (33.1%) complained of dizziness at their first visit, and 95 (64.2%) yielded abnormal results in more than one of the vestibular function tests (Table 1, Fig. 2). Canal paresis on the caloric test was revealed in 43 cases (29.1%) and abnormal cVEMP in 60 patients (40.5%). vHIT abnormalities of the anterior, horizontal and posterior SCCs were observed in 12.8%, 16.9%, and 18.9% of enrolled patients, respectively. The mean hearing gain in the 3 months from onset was 23.8 ± 23.7 dB HL. Complete recovery of normal hearing was observed in 71 patients (48.0%) (Table 1).

**Table 1:** Demographic and clinical characteristics of the study population.

Variable	Patients (N = 148)
Sex	
Male/Female	62(41.9)/86(58.1)
Age	49.7 ± 13.9
Underlying Disease	
Hypertension,No. (%) with data	36 (24.3)
Diabetes, No. (%) with data	18 (12.2)
Cardio vascular disorder, No. (%) with data	3 (2.0)
Affected Side	
Right, No. (%) with data	70 (47.3%)
Left, No. (%) with data	78 (52.7%)
Associated symptom	
Dizziness/Vertigo	49 (33.1%)
Tinnitus	97 (65.5%)
Ear fullness	69 (46.6%)
Onset of treatment, Mean (SD), days	5.2 ± 6.2
Initial hearing threshold, Mean (SD), dB	62.2 ± 29.5
Treatment method	
High dose oral steroid, No. (%) with data	57 (38.5%)
Oral steroid + IT dexamethasone*, No. (%) with data	91 (61.5%)
Abnormal Caloric test (Canal paresis), No. (%) with data	43 (29.1%)
Abnormal VEMP, No. (%) with data	60 (40.5)
Video head impulse test, No. (%) with data	
Abnormal gain in any semi-circular canal	51 (34.5%)
Abnormal Anterior. canal	19 (12.8%)
Abnormal Horizontal canal	25 (16.9%)
Abnormal Posterior canal	28 (18.9%)
Hearing gain, Mean (SD), dB	23.8 ± 23.7
Hearing recovery, No. (%) with data	
Complete recovery^a^	71 (48.0%)
Partial recovery^b^	28 (18.9%)
Slight improvement^c^	27 (18.2%)
No improvement^d^	22 (14.9%)

*IT: Intratympanic injection.

Complete recovery: a final hearing threshold <25 dB HL.

Partial recovery: final hearing threshold of 26–45 dB HL, and >15 dB of hearing gain.

Slight improvement: final hearing threshold >46 dB HL and <15 dB of hearing gain.

No improvement: final hearing threshold >76 dB HL, or <15 dB of hearing gain.

### Known prognostic factors for hearing recovery

Univariable analyses of previously-suggested prognostic factors for hearing outcomes are shown in Table 2. Younger age and lower initial hearing threshold were associated with complete recovery (P = 0.012, P < 0.001). Hearing recovery was negatively related to canal paresis (P = 0.001) and to vertigo (P = 0.003). Other parameters including sex, underlying disease and cVEMP abnormalities did not show any significant relation to recovery.

**Table 2:** Clinical parameters associated with hearing recovery in idiopathic sudden sensorineural hearing loss.

Parameter	Univariable Analysis			Multivariable Analysis	
	Complete Recovery^a^ (n = 71)	Incomplete Recovery (n = 77)	*P*^b^	Exp(B)	*P*^c^
Sex (men: women)	27:44	35: 42	0.406		
Age (mean)	46.7 ± 14.5	52.4 ± 12.9	**0.012**	**1.040**	**0.011**
Onset of treatment (days)	4.4 ± 4.5	6.0 ± 7.3	0.117		
Underlying disease					
Hypertension	15 (20.8%)	21 (27.6%)	0.445		
Diabetes	6 (8.3%)	12 (15.8%)	0.215		
Cardiovascular	0	3 (3.9%)	0.246		
Associated symptoms					
Vertigo	15 (21.1%)	34 (44.2%)	**0.003**		
Tinnitus	50 (70.4%)	48 (62.3%)	0.385		
Ear fullness	36 (50.7%)	33 (42.9%)	0.410		
Initial hearing level (dB)	47.6 ± 21.2	75.7 ± 29.8	**<0.001**	**1.045**	**<0.001**
Hearing gain (dB)	32.7 ± 21.1	15.0 ± 22.6	**<0.001**		
Canal paresis (%) on caloric test^d^	16.0 ± 16.9	30.2 ± 32.2	**0.001**	**1.023**	**0.023**
Abnormal CP on caloric test^e^	13 (18.3%)	30 (39.0%)	**0.007**		
Abnormal cVEMP	26 (36.6%)	34 (44.2%)	0.351		
Video Head impulse test					
Abnormal vHIT, AC	9 (12.5%)	10 (13.2%)	1.000		
Abnormal vHIT, HC	7 (9.7%)	18 (23.7%)	**0.031**	1.313	0.667
Abnormal vHIT, PC	4 (5.6%)	24 (31.2%)	**<0.001**	**3.690**	**0.047**

^a^Complete recovery of hearing defined as pure tone threshold 25 dB or better.

^b^Univariable logistic regression analysis.

^c^Multivariable logistic regression analysis.

^d^Canal paresis (%) according to Jongkees’ formula^17^.

^e^Number of patients showing abnormal canal paresis (<25%) on the caloric test.

Statistically significant (P < 0.05) values are highlighted in bold.

### Prognostic value of vHIT abnormality for hearing recovery

**Figure 3:**
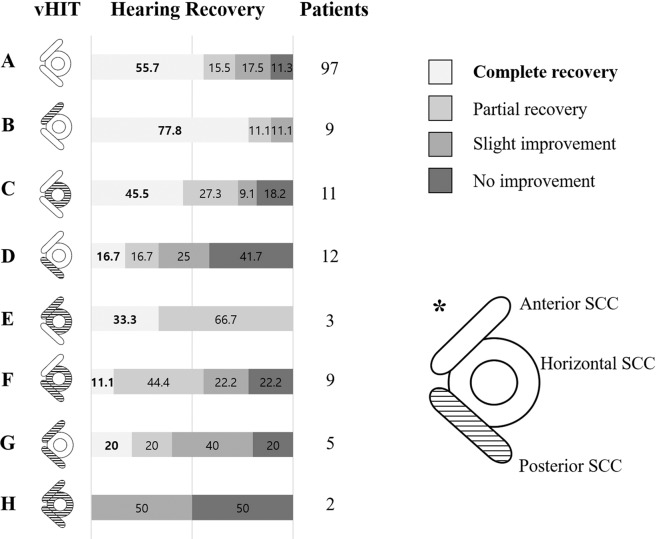
Type of semicircular canal involvement in the video head impulse test and corresponding hearing outcomes. *The striped area indicates that the gain of vHIT in the corresponding canal decreased (e.g. abnormal gain in the posterior SCC). vHIT; video head impulse test, Patients; number of patients, SCC; semicircular canal.

Abnormal vHIT gain in any of the SCCs was seen in fifty-one patients (34.5%) (Table 1). Hearing outcomes according to the vHIT results are shown in Fig. 3. Of the 97 patients with normal vHIT results, 54 (55.7%) recovered normal hearing (Fig. 3A) whereas none of the patients with vHIT abnormalities in all SCCs recovered normal hearing (Fig. 3H).

Univariable analyses showed that abnormal vHIT results in the horizontal SCC (P = 0.031) and posterior SCC (P < 0.001) were significantly related to poor recovery (Table 2).

In a multivariable analysis including the objective clinical factors that were statistically significant in the univariable analysis, significant predictive indicators for incomplete recovery were age (OR 1.040, P = 0.011), poorer initial hearing level (OR 1.045, P < 0.001), canal paresis on the caloric test (OR 1.023, P = 0.023) and abnormal vHIT gain in the posterior SCC (OR 3.690, P = 0.047) (Table 2). Low vHIT gain in the posterior SCC had specificity of 94.4% and PPV of 85.7% in predicting incomplete recovery of hearing (Table 3).

**Table 3:** Sensitivity, specificity, positive predictive value and negative predictive value of decreased video head impulse test gain in the posterior semicircular canal in predicting incomplete hearing recovery.

		Observed Hearing Outcome			
		Incomplete Recovery	Complete Recovery		
Predicted	Abnormal PSCC vHIT	**24**	**4**	Positive predictive value	**85.7%**
	Normal PSCC vHIT	**53**	**67**	Negative predictive value	56.7%
		Sensitivity	Specificity		
		31.2%	**94.4%**		

## Discussion

In the present study, we assessed the prognostic value of SCC involvement in ISSNHL using the vHIT. The results can be summarized as follows: (1) Various patterns of vHIT abnormality with SCC involvement were observed with or without subjective dizziness, (2) Multivariable analysis showed that incomplete recovery of hearing was significantly associated with decreased vHIT gain in the posterior SCC (OR 3.690, P = 0.047), worse initial hearing level at onset (OR 1.045, P < 0.001), age at onset (OR 1.040, P = 0.011), and canal paresis on the caloric test (OR 1.023, P = 0.023), (3) In our preliminary data, decreased vHIT gain in the posterior SCC had specificity of 94.4%, sensitivity of 31.2%, PPV of 85.7% and NPV of 56.7% in predicting incomplete recovery.

Since its introduction in 1988 by Halmagyi and Curthoys, the head impulse test has been used to evaluate the functioning of SCCs^22^. The video method of examination, vHIT, is a noninvasive and quantitative method assessing each of the six SCCs individually^14^. In the vHIT, the adequacy of the VOR is usually measured by the gain, the ratio of the area under the eye velocity curve to the area under the head velocity curve during small, fast, passive unpredictable head impulses^14^. A VOR gain of <0.7 is usually considered as identifying a deficient SCC^14^. The vHIT has been widely applied to evaluate SCC function, especially in peripheral vestibular disorders and some central lesions^14,23–28^. In this study, we used it to assess vestibular involvement in ISSNHL.

Prognostic indicators for ISSNHL have been widely investigated^1,29^. The well-known factors include age at onset, severity of initial hearing loss and time to initial treatment^1^. In our study, older age and poor initial hearing level were significantly associated with incomplete recovery of hearing (Table 2), in agreement with previous findings. Time to onset of treatment did not have any significant effect because the majority of our patients (140/148, 94.6%) were seen within 2 weeks of onset, which is considered the responsive period for treatment^2^. Average time to onset of treatment was 5.2 days (Table 1).

Vestibular involvement in SSNHL has been an interesting issue^7,9,30^. Subjective imbalance or vertigo is associated with poor hearing recovery^1,30^. In our study, a third of the patients complained of dizziness (Table 1, Fig. 2), and this was significantly related to incomplete recovery (Table 2) We noted, however, that not all patients who showed decreased vestibular function in the tests complained of vertigo, and those with subjective dizziness did not always have vestibular function abnormalities (Fig. 2).

In terms of evaluation of vestibular function, Yu et al., in their systemic review and meta-analysis, reported that about half of SSNHL patients had abnormal vestibular function in more than one of the tests including the caloric test, cVEMP and oVEMP^30^. They also postulated that damage to the horizontal SCC (caloric test) and saccule (cVEMP) was an important factor for hearing recovery, and that the extent of vestibular damage was related to the prognosis of hearing loss^30^. In addition, Fujimoto et al. reviewed the results of vestibular function tests in ISSNHL patients with vertigo to assess the extent of vestibular lesions^7^. They showed that the vestibular end organs close to the cochlea tended to be preferentially affected: there was involvement of saccule in 64%, of the horizontal SCC in 52% and of the utricle in 43% of the cases^7^. In our study, about 2/3 of the patients gave abnormal results in vestibular function tests. As in previous reports, the saccule was most often affected (60/148, 40.5%) followed by the horizontal SCC, based on abnormal caloric responses (43/148, 29.1%) (Table 1). Worse canal paresis (%) in the caloric test was significantly associated with incomplete hearing recovery (OR 1.023, P = 0.023) (Table 2) as in previous work^6–8^. However, the extent of SCC damage was not closely correlated with hearing outcome, especially in patients with partial involvement (Fig. 3). In terms of horizontal SCC involvement, there were discrepancies between abnormalities in the caloric test (43/148, 29.1%) and the vHIT (25/148, 16.9%) (Table 1). These could be explained by the contribution of the vertical canals to the caloric response, as shown in previous research^31^.

Interestingly, low vHIT gain in the posterior SCC was found to be an independent parameter affecting hearing recovery (OR 3.690, P = 0.047) (Table 2). Among the patients who showed complete recovery, sixty-seven (67/71, 94.4%) had normal vHIT gains in the posterior SCC (Table 3), whereas of those with abnormal vHIT results in the posterior SCC, only four (4/28, 14.3%) recovered completely (Fig. 2). In addition, eleven of those with abnormal vHIT results (11/28, 39.3%) had normal caloric responses, and eight (8/28, 28.6%) did not complain of dizziness, which means that those patients may be considered as having normal vestibular function if vHIT was not performed.

In terms of the inflammatory origin of ISSNHL, the involvement of adjacent vestibular organs can be explained by the flow of endolymphatic fluid within the inner ear, as well as the proximity of the vestibulocochlear nerve, and vestibular involvement can vary between individual patients. Empirical corticosteroid therapy may be expected to play a crucial role in such cases, as well as where viral infection and autoimmunity are implicated.

It has also been suggested that certain patterns of vestibular involvement point to a vascular etiology for ISSNHL, as opposed to an inflammatory cause. In particular, compromise of the common cochlear artery can induce sudden hearing loss with isolated posterior SCC hypofunction (Fig. 1)^9^. Rambold et al. described a subgroup of patients with a distinct lesion pattern specifically involving the posterior SCC and cochlea^9^. In our study, twelve patients (12/148, 8.1%) showed this pattern (Fig. 3D). To compare this specific group with others showing anterior or horizontal SCC involvement (Fig. 3B,C), a post-hoc analysis was performed. Hearing outcomes were notably poorer in patients with posterior SCC involvement compared to involvement of other individual canals (P = 0.028 by Fisher’s exact test). Based on the results of an animal study showing that ischemia of 30 minutes or longer induces irreversible cochlear damage^32^, the vulnerability of the cochlea to ischemia might offer a possible explanation for this outcome.

In the present study, multivariable analysis showed that risk factors for incomplete recovery were older age, low initial hearing level, canal paresis on the caloric test and abnormal vHIT gain in the posterior SCC (Table 2). Posterior SCC involvement had the highest odds ratio of the clinical factors considered (OR = 3.690). It seems that the functioning of the posterior SCC reflects the severity of cochlear damage better than other indicators. Vascular supply, as mentioned above, as well as the close proximity of the cochlear nerve to the vestibular nerve fibers innervating the posterior SCC, may provide a rationale for this result (Fig. 1).

To the best of our knowledge this is the first study assessing the prognostic value of SCC involvement in ISSNHL using the vHIT. We assessed all ISSNHL patients, regardless of subjective dizziness. In addition, the inclusion criteria included normal contra-lesional hearing to alleviate possible debate about outcome determination. The evaluation of vestibular function, including each SCC, provided distinctive results pointing to a new prognostic indicator of hearing recovery. Unfortunately, oVEMP, which assesses utricular function, was not included in our analysis due to a lack of reliable data. Although we reviewed consecutive ISSNHL patients who were managed with a uniform treatment protocol during the enrollment period, there remains a possibility that biases may have arisen from the retrospective nature of the study. Hopefully, a future prospective study including all available vestibular function tests in a large number of patients will give further insight into the role of vestibular function in ISSNHL. Apart from providing prognostic information, we expect future studies to help develop individual treatment strategies to improve outcomes in ISSNHL.

## Conclusion

Abnormal vHIT gain in the posterior SCC is probably a specific prognostic factor indicating incomplete hearing recovery in ISSNHL.

## Data Availability

Anonymized data will be shared on request from any qualified investigator for the purpose of replicating procedures and results.
